# Editorial: Recent Advances in Microbial Biotechnology for the Food Industry

**DOI:** 10.3389/fmicb.2021.746636

**Published:** 2021-09-27

**Authors:** Dan Cristian Vodnar, Joachim Venus, Laurent Dufossé

**Affiliations:** ^1^Faculty of Food Science and Technology, University of Agricultural Sciences and Veterinary Medicine of Cluj-Napoca, Cluj-Napoca, Romania; ^2^Department of Bioengineering, Leibniz Institute for Agricultural Engineering and Bioeconomy (ATB), Potsdam, Germany; ^3^Université de la Réunion, CHEMBIOPRO Lab, ESIROI agroalimentaire, Saint-Denis, France

**Keywords:** *in situ* microbial fermentation, value-added compound, flavor, omega-3, Lactobacillus, fructo-oligosaccharide, immobilized microbial enzyme, food waste valorization

The progress made in the food industry by the development of applicative engineering and biotechnologies is impressive and many of the advances are oriented to solve the world crisis in a constantly growing population (Călinoiu et al., 2018). Microorganisms possess an impressive role in supporting life, either separate or in consortia, by producing numerous useful molecules (Mitrea et al., 2017; Martău et al., 2021). Therefore, the goal of the present Research Topic was to tackle the *in situ* microbial production by *de novo* biosynthesis of value-added compounds, such as flavors (vanillin), omega 3 (DHA), organic acids, etc., emergent for the food industry, and their characterization. The accent was on the substrate used, as well on the performance of the microbial process, and different ways for process downstream.

Several industries have exploited the biochemical capability of microorganisms to synthesize, metabolize and transform valuable substances as one of the most popular topics deals with the increasing demand for natural aromas, colorants, flavoring agents and food additives. According to the literature, besides the extraction and isolation from natural material sources, which is not able to supply the demand, has a low yield and is not cost-effective (Zhou et al., 2019), the natural aromas are generated also by enzymatically biotransformation of precursors and *de novo* synthesis by microorganisms (Paulino et al., 2021). Moreover, the microbial engineering generates impressive yields and satisfied the consumers' needs as they are considered “natural.” The biotransformation approach requires the presence of specific enzymes, whereas immobilized enzymes are the most efficient toward industrial molecules bioproduction. In the study of Varga et al., one of the contributing article to the present Special Issue, the enzymes immobilization strategy used was based on the recombinant poly-His-tag fused enzymes on metal-chelated carriers for reducing the carbonyl compounds to their corresponding alcohols. The authors succeded to convert 61% of the acetophenone to (S)-1-phenylethanol by recombinant alcohol dehydrogenase (RrADH) from *Rhodococcus ruber*, and 88% of the *trans-*2-hexenal to *trans-*2-hexenol by recombinant *Saccharomyces cerevisiae* alcohol dehydrogenase (ScADH1) with simultaneous NADH regeneration by recombinant *Candida boidinii* formate dehydrogenase (FDH).

Industrial by-products are certainly an economical source of natural compounds (Vodnar et al., 2017; Mitrea et al., 2020; Ştefănescu et al., 2020) (e.g., polyphenols, carotenoids, sterols, tocopherols, vitamins, or dietary fiber), and recovery and recycling of these wastes via microbial route may contribute toward the sustainability of the industrial and food sectors. Waste bioconversion represents a supportive strategy in the current waste crisis and massive pollution of our planet. Moreover, their bioconversion may contribute to obtaining several value-added compounds, such as organic acids, omega 3, short-chain fatty acids, flavor agents, functional exopolysaccharides, nutraceuticals. In the Special Issue-derived contributing study of Patel et al., volatile fatty acids generated during the anaerobic digestion of food waste were used as a feedstock for specific microalgae in order to produce valuable lipids, such as polyunsaturated fatty acids (PUFA) and saturated fatty acids (SFA). In the another contributing article, Fan et al. successfully purified the functional fructo-oligosaccharides (FOS) from crude preparations by employing a probiotic bacteria fed-batch fermentation process demonstrating the selective consumption of carbon sources by probiotic microorganisms in a mixture of monosaccharides and oligosaccharides substrate. The use of microorganisms for value-added compounds production has received increasing attention, as complex natural products can be delivered from inexpensive raw materials on industrial scale (Călinoiu et al., 2019), like is the case of fermented dairy products. In the mini-review of Widyastuti et al. the lactobacilli (genus *Lactiplantibacillus*) health-promoting effects in dairy fermentation was critically revised, whereas the bioactive peptides production by lactobacilli and their probiotic status were demonstrated to be the most important features in human health.

On the other side, a major attention in food industry is focused on food poisoning with has direct impact on human health. Among the microorganisms responsible for food poisoning, *Staphylococcus aureus* is one of the most common, whereas the sequence typing analysis for identifying the isolates and the enterotoxins produced are the latest topics explored in this field. In the study of Lv et al., the molecular characteristics, involving genotypes, resistance profile and enterotoxigenic status of *Staphylococcus aureus*, from food samples and food poisoning outbreaks in Shijiazhuang, China, was investigated. This contributing study explained the prevalence, contamination and transmission of foodborne *S. aureus* in food infections based on the epidemic characteristics.

The present Research Topic encouraged the submission of high-quality research articles and reviews covering the most recent advances in microbial biotechnology for the food industry. Building upon findings published in the *recent Topic Microbial Biotechnology Providing Bio-based Components for the Food Industry*, we wished to emphasize the industrial application potential, in particular the current progress, actual concerns in the biotechnological field, and success of very recent technologies, such as bioengineering and fermentation.

## Author Contributions

DV and LD designed and wrote the editorial with contributions from JV. All authors contributed to the article and approved the submitted version.

## Conflict of Interest

The authors declare that the research was conducted in the absence of any commercial or financial relationships that could be construed as a potential conflict of interest.

## Publisher's Note

All claims expressed in this article are solely those of the authors and do not necessarily represent those of their affiliated organizations, or those of the publisher, the editors and the reviewers. Any product that may be evaluated in this article, or claim that may be made by its manufacturer, is not guaranteed or endorsed by the publisher.
